# Dataset on the changes of neutrophils treated with retinoic acid

**DOI:** 10.1016/j.dib.2017.03.032

**Published:** 2017-03-22

**Authors:** Sanjeeb Shrestha, Shin-Yeong Kim, Young-Jin Yun, Jun-Kyu Kim, Jae Man Lee, Minsang Shin, Dong-Keun Song, Chang-Won Hong

**Affiliations:** a Department of Pharmacology, College of Medicine, Hallym University, Chuncheon 24252, Republic of Korea; b Department of Physiology, School of Medicine, Kyungpook National University, Daegu 41944, Republic of Korea; c Department of Biochemistry & Cell Biology, School of Medicine, Kyungpook National University, Daegu 41944, Republic of Korea; d Department of Microbiology, School of Medicine, Kyungpook National University, Daegu 41944, Republic of Korea

**Keywords:** Neutrophil, Retinoic acid, Phenotype, Hypersegmentation

## Abstract

The data presented in this article are related to the research article entitled “Retinoic acid induces hypersegmentation and enhances cytotoxicity of neutrophils against cancer cells” (S. Shrestha, S.Y. Kim, Y.J. Young, J.K. Kim, J.M. Lee, M. Shin, D.K. Song, C.W. Hong, 2017) [1]. This article complements the potential of retinoic acid to induce changes in effector function of human neutrophils. Here the datasets describe the rate of apoptosis, changes in numbers of nuclear lobes, and the expressions of surface markers in human neutrophils in presence or absence of retinoic acid. The tumor growth in recipient mice with adoptive transfer of retinoic acid-treated neutrophils was evaluated. The included data is made publicly available to criticism and extended analysis.

**Specifications Table**

**Table:** 

Subject area	*Biology*
More specific subject area	*Immunology, Innate immunity, tumor immunology*
Type of data	*Text and figures*
How data was acquired	*Neutrophils were isolated from either healthy volunteer or bone marrow of mice and stimulated with retinoic acid. The changes were analyzed using FACS (BD FACS Calibur), Microscopic image (Nikon Eclipse Ti-E), and measurement of in vivo tumor growth*
Data format	*Analyzed*
Experimental factors	*Neutrophils were exposed to retinoic acid*
Experimental features	*Apoptosis, nuclear segmentation, and surface marker expressions of neutrophils were determined. Tumor growth in recipient mice was examined.*
Data source location	*Kyungpook National University, Daegu, Republic of Korea,*
Data accessibility	*Data are presented in this article*

**Value of the data**

• The data highlights the changes in the function of human neutrophils after treatment with retinoic acid.
• The data can be useful to compare the rate of apoptosis in neutrophils induced by retinoic acid.
• The data shows changes in the nuclear morphology and surface expression markers induced by retinoic acid treatment and the effect of adoptive transfer of retinoic-acid-treated donor neutrophils on tumor growth in recipient mouse.
• The data can be useful for other researchers investigating the effects of retinoic acid on neutrophils.

## 1 Data

**Fig. 1:**
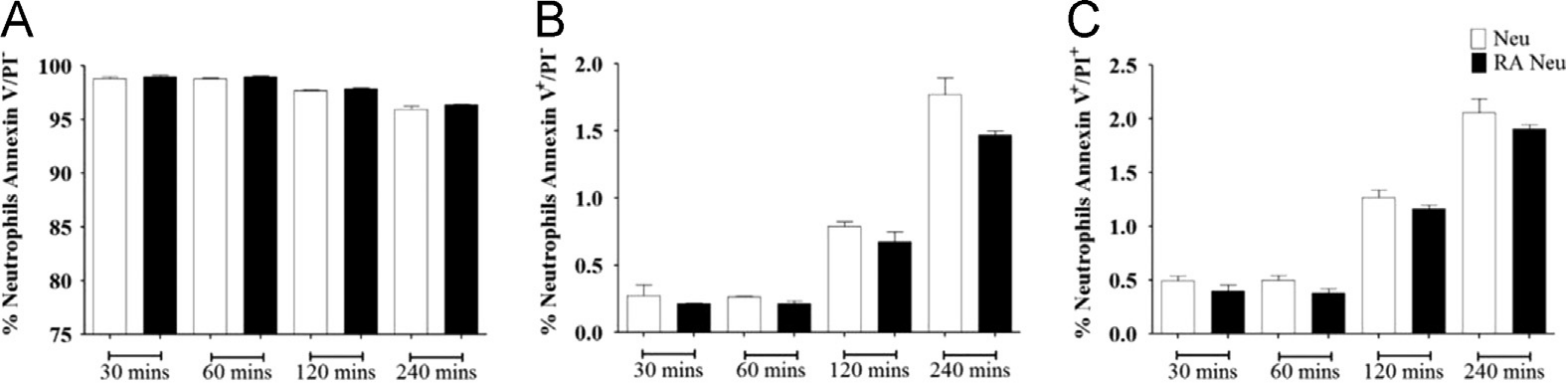
Effect of retinoic acid on apoptosis of neutrophils. Neutrophils (1× 10^6^ cells/ml) exposed to retinoic acid (100 nM) for indicated the time and neutrophil survival was examined by annexin V/propidium iodide (PI) staining. Apoptosis (annexin V-positive only) and necrosis (double positive for annexin-V and PI) rates in neutrophils. (A) The percentage of live neutrophils (annexin V-PI double negative), (B) The percentage of apoptotic neutrophils (annexin V-positive only), (C) The percentage of necrotic cells (annexin V-PI double positive). All results are shown as means±SEMs (*n*=2).

**Fig. 2:**
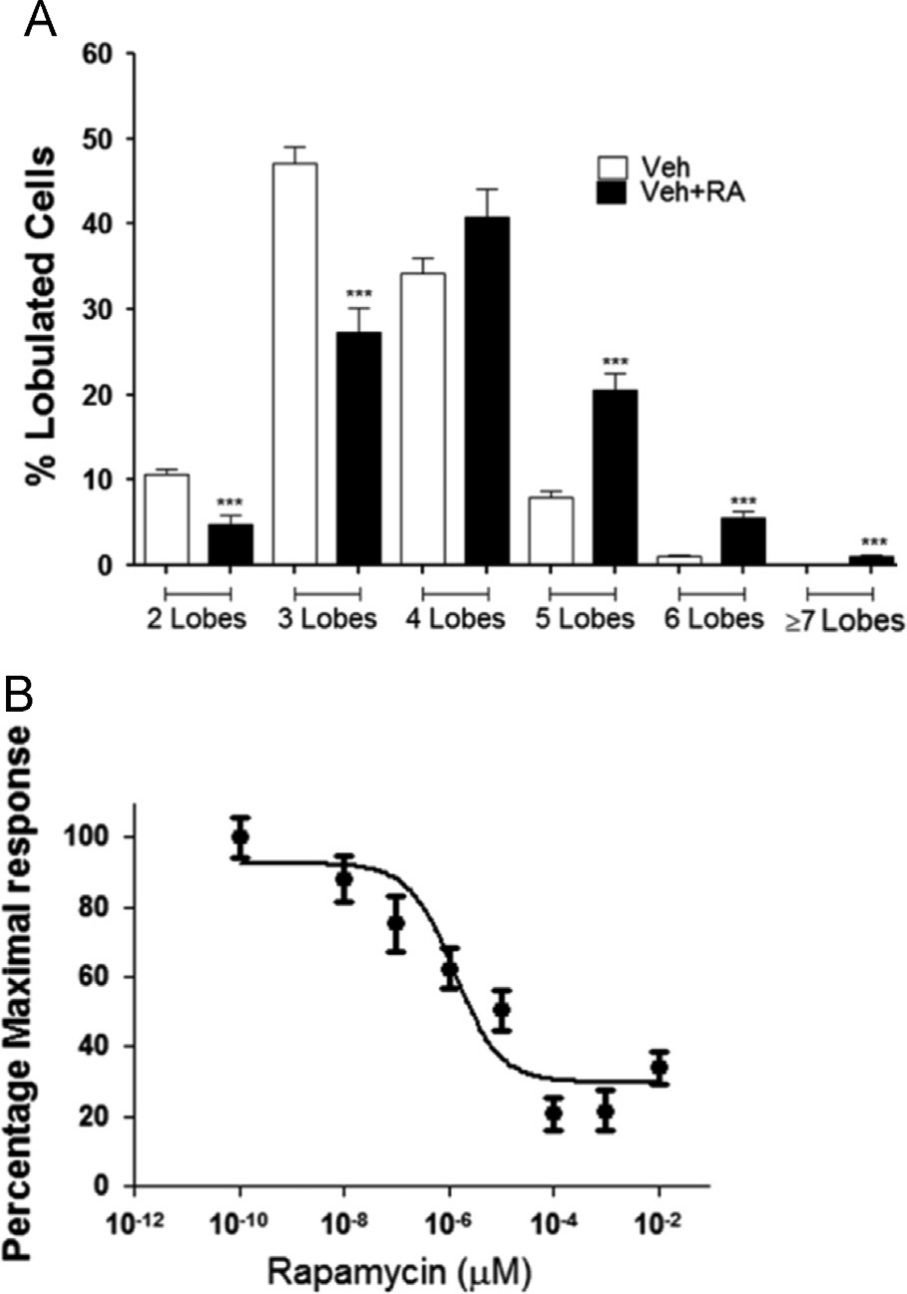
Changes in nuclear lobe count in neutrophils treated with retinoic acid. (A) Distributions of nuclear lobes in the vehicle- and retinoic acid-treated neutrophils. The difference was analyzed using both student *t*-test and ANOVA (*n*=8). *** *p*<0.001 compared to control groups (Veh) (*n* =8 for each group). (B) The effect of rapamycin on the retinoic acid-induced hypersegmentation. Neutrophils were incubated with rapamycin at indicated concentrations and further stimulated with retinoic acid (100 nM). Maximal response denotes lobe counts of retinoic acid-treated neutrophils (*n* = 3 for each group). All results are shown as means±SEMs.

**Fig. 3:**
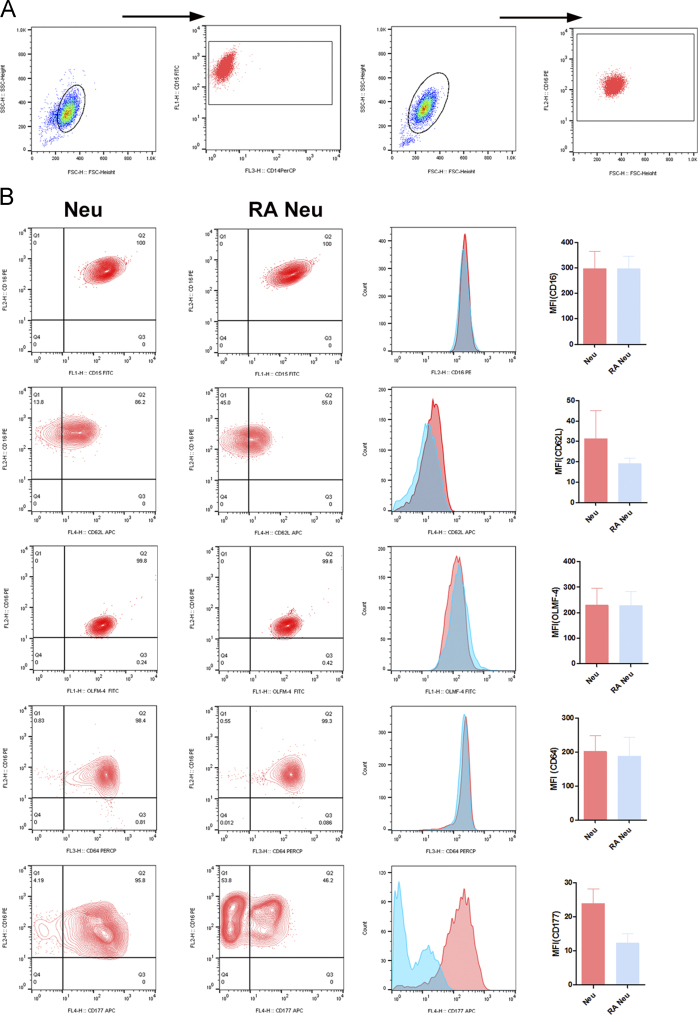
Expression of surface receptors in neutrophils treated with retinoic acid. Vehicle- and retinoic acid-treated neutrophils were assayed for expressions of surface receptors and adhesion molecules by flow cytometry. (A) Gating strategy for neutrophils. Neutrophils were considered as CD14^−^ CD 15^+^ CD16^+^ cells. (B) Left, representative flow cytometric dot plot of vehicle-treated neutrophils (Neu) and retinoic acid-treated neutrophils (RA Neu). Middle, histograms for each surface markers. Right, the mean fluorescence intensity of each surface markers. All results are shown as means±SEMs (*n*=6 for each group).

**Fig. 4:**
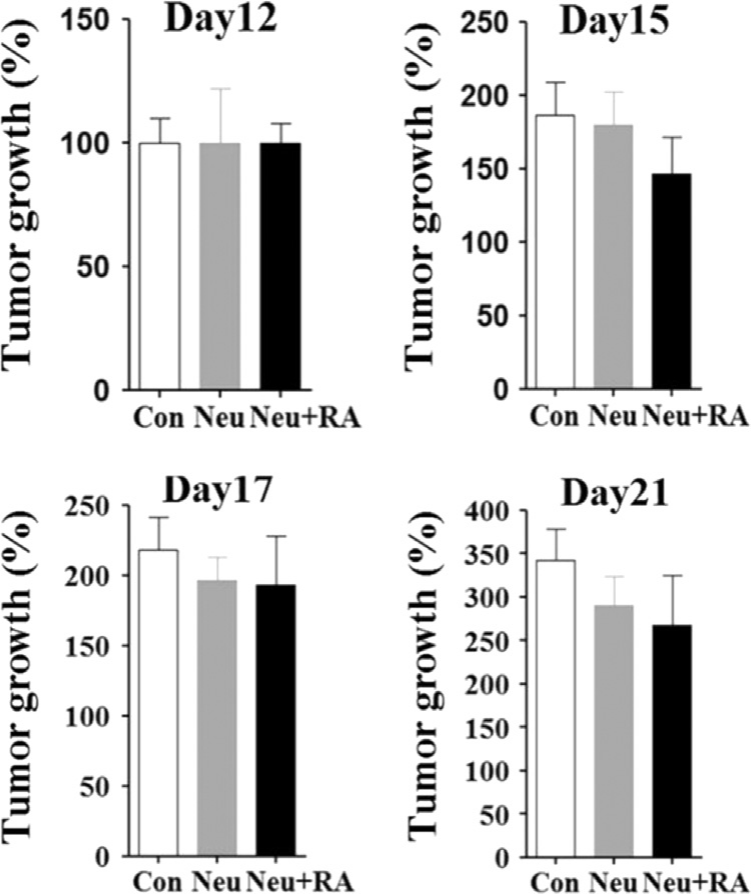
Tumor growth in recipient mice with adoptive transfer of retinoic acid-treated neutrophils. Neutrophils were isolated from bone marrow of naïve mice and further treated with retinoic acid (100 nM). Neutrophils were intratumorally injected to recipient tumor bearing mice on day 13 and 15. The relative percentages of tumor growth compared to vehicle-injected mice were calculated. All results are shown as mean±SEM (*n*=4 mice for each group).

The data includes information regarding the effect of retinoic acid on neutrophils complementing the previous study [1]. The apoptosis rates of neutrophils exposed to either retinoic acid (100 nM) or vehicle were examined using annexin/ propidium iodide (PI) fluorescence-activated cell sorting (FACS) staining (Fig. 1). The percentages of living (annexinV^−^/PI^−^ cells, Fig. 1A), apoptotic (annexinV^+^/PI^−^ cells, Fig. 1B), and necrotic (annexinV^+^/PI^+^ cells, Fig. 1C) neutrophils were described. Next, the mean lobe counts of retinoic acid-treated neutrophils were examined using hemacolor staining (Fig. 2). The lobe counts of neutrophils were described (Fig. 2A), and the effect of rapamycin on the lobe counts of retinoic acid-treated neutrophils were described as IC_50_ (Fig. 2B). The expression of surface markers in retinoic acid-treated neutrophils were examined using FACS analysis (Fig. 3). The gating strategies for evaluation of neutrophil surface markers were described (Fig. 3A). The surface expression of CD62L (L-selectin), CD177 (NB1, a glycosyl-phosphatidylinositol-linked N-glycosylated cell surface glycoprotein), CD16 (FcγRIIIa), CD64 (FcγRI), and OLMF-4 (Olfactomedin) were analyzed (Fig. 3B). Finally, the intratumoral adoptive transfer of retinoic acid-treated neutrophils from donor mouse into the recipient tumor-bearing mice were examined (Fig. 4).

## 2 Experimental design, materials and methods

### 2.1 Neutrophil isolation

Human blood experiments were approved by the Institutional Research Board of Hallym University and Kyungpook National University. Neutrophils were isolated from venous blood by double density gradient method using histopaque 1077 and 5% dextran [2].

### 2.2 Apoptosis detection

Neutrophils (1×10^6^ cells/ml) were treated with retinoic acid (100 nM) for the indicated time. Neutrophils were collected and stained with annexin V/propidium iodide following instructions of apoptosis detection kit. Then cell apoptosis was detected using flow cytometer.

### 2.3 Wright–Giemsa staining for mean lobe count

Hypersegmentation in neutrophils was determined by Wright–Giemsa staining. Isolated neutrophils (2×10^6^) were treated with retinoic acid (100 nM, Sigma-Aldrich) for 4 h. Cells were then cytospun and stained with hemacolor stain (Merck Millipore) and mean lobe counts were determined.

### 2.4 Flow cytometry

Neutrophils were fixed and then stained. Antibodies targeted against different surface receptors: CD15, CD14, CD16 and CD64 and adhesion molecules: CD177, CD62L, and OLMF-4: FITC conjugate were used. The cells were washed and dissolved in staining buffer. Data acquisition was done using flow cytometer (BD FACS Calibur) and data analyzed by Flowjo.

### 2.5 Adoptive transfer of retinoic acid-treated neutrophils

Neutrophils from naïve mice were harvested by positive selection using Ly6G^+^ MACS kit. The neutrophils were treated with retinoic acid (100 nM) for 4 h. Recipient BALB/c mice (five weeks old, female) were injected with 1×10^5^ 4T1 cells in the right leg. Then, recipient tumor-bearing mice were administered retinoic acid treated neutrophils (5×10^6^ cells) on day 13 and 16 post tumor injection. Tumor growth was measured every three days until day 21.

### 2.6 Statistical analysis

All statistical analyses were performed using GraphPad prism 5.0 (Graphpad software). AVOVA analysis or two-tailed Student׳s *t*-test were performed to determine the significance.

## References

[1] Shrestha S., Kim S.Y., Youn Y.J., Kim J.K., Lee J.M., Shin M., Song D.K., Hong C.W. (2017). Retinoic acid induces hypersegmentation and enhances cytotoxicity of neutrophils against cancer cells. Immunol. Lett..

[2] Shrestha S., Noh J.M., Kim S.Y., Ham H.Y., Kim Y.J., Yun Y.J., Kim M.J., Kwon M.S., Song D.K., Hong C.W. (2016). Angiotensin-converting enzyme inhibitors and angiotensin II receptor antagonist attenuate tumor growth via polarization of neutrophils toward an antitumor phenotype. Oncoimmunology.

