# Supplementary material for: Dataset on the changes of neutrophils treated with retinoic acid
Source: Data Brief. 2017 Mar 22;12:97–102. doi: 10.1016/j.dib.2017.03.032 (PMC5382026; doi:10.1016/j.dib.2017.03.032)
Supplement: Supplementary file 1 — Supplementary material [file mmc1.pdf]

16 Jan, 2016

Dear Editor,

Here we certify that all authors have seen and approved the manuscript being submitted and also declare that none of the author have a financial interest related to this work.

Sincerely,

Chang-Won Hong, M.D.,Ph.D.

Department of Physiology, School of Medicine, Kyungpook National University

680 Gukchaeborang-ro, Daegu 41944, Republic of Korea.

Tel 82-53-420-4812

E-mail: cwhong@knu.ac.kr

Disclosure:

I have no relevant financial interest related to this work

Sanjeeb Shrestha, M.S.

Department of Pharmacology, College of Medicine, Hallym University, Chuncheon,

24252, Republic of Korea.

200-702, Republic of Korea

Tel: +82-33-248-3291

E-mail: sanjeebs2012@gmail.com

Disclosure:

I have no relevant financial interest related to this work

Sanjeeb

Shin-Yeong Kim, M.S.

Department of Pharmacology, College of Medicine, Hallym University, Chuncheon, 24252, Republic of Korea.

E-mail: kkdnsy@naver.com

Disclosure:

I have no relevant financial interest related to this work

김신영

Young-Jin Youn

Department of Pharmacology, College of Medicine, Hallym University, Chuncheon, 24252, Republic of Korea.

E-mail: yunyj1203@gmail.com

Disclosure:

I have no relevant financial interest related to this work

Jun-Kyu Kim

Department of Physiology, School of Medicine, Kyungpook National University, Daegu, 41944, Republic of Korea.

E-mail: rlawnsrb1517@naver.com

Disclosure:

I have no relevant financial interest related to this work

Jae Man Lee

Department of Biochemistry & Cell Biology, School of Medicine, Kyungpook National University, Daegu, 41944, Republic of Korea.

E-mail: jaemanlee@knu.ac.kr

Disclosure:

I have no relevant financial interest related to this work

Minsang Shin

Department of Microbiology, School of Medicine, Kyungpook National University, Daegu, 41944, Republic of Korea.

E-mail: shinms@knu.ac.kr

Disclosure:

I have no relevant financial interest related to this work

Dong-Keun Song, M.D.,Ph.D.

Department of Pharmacology, College of Medicine, Hallym University, Chuncheon, 24252, Republic of Korea.

E-mail: dksong@hallym.ac.kr

Disclosure:

I have no relevant financial interest related to this work
